# Exploring the nexus between fatigue, body composition, and muscle strength in hemodialysis patients

**DOI:** 10.1186/s40001-024-01852-1

**Published:** 2024-05-03

**Authors:** Rui Shi, Jia-xin Zhu, Li Zhu, Wen-man Zhao, Huai Li, Qi-chun Chen, Hai-feng Pan, De-guang Wang

**Affiliations:** 1 Department of Nephrology, The Second Affiliated Hospital of Anhui Medical University, 678 Furong Road, Hefei, China; 2 Institute of Kidney Disease, Inflammation & Immunity Mediated Diseases, The Second Affiliated Hospital of Anhui Medical University, 678 Furong Road, Hefei, China; 3 Department of Radiology, The Second Affiliated Hospital of Anhui Medical University, 678 Furong Road, Hefei, China; 4 Department of Epidemiology and Biostatistics, School of Public Health, Anhui Medical University, 81 Meishan Road, Hefei, China

**Keywords:** Fatigue, Intermuscular adipose tissue, Paraspinal muscle, Hemodialysis

## Abstract

**Background:**

Fatigue is a relatively prevalent condition among hemodialysis patients, resulting in diminished health-related quality of life and decreased survival rates. The purpose of this study was to investigate the relationship between fatigue and body composition in hemodialysis patients.

**Methods:**

This cross-sectional study included 92 patients in total. Fatigue was measured by Functional Assessment of Chronic Illness Therapy – Fatigue (FACIT-F) (cut-off ≤ 34). Body composition was measured based on quantitative computed tomography (QCT), parameters including skeletal muscle index (SMI), intermuscular adipose tissue (IMAT), and bone mineral density (BMD). Handgrip strength was also collected. To explore the relationship between fatigue and body composition parameters, we conducted correlation analyses and binary logistic regression.

**Results:**

The prevalence of fatigue was 37% (n = 34), abnormal bone density was 43.4% (*n* = 40). There was a positive correlation between handgrip strength and FACIT-F score (*r* = 0.448, *p* < 0.001). Age (*r* = − 0.411, *p* < 0.001), IMAT % (*r* = − 0.424, *p* < 0.001), negatively associated with FACIT-F score. Multivariate logistic regression analysis shows that older age, lower serum phosphorus, higher IMAT% are associated with a high risk of fatigue.

**Conclusion:**

The significantly increased incidence and degree of fatigue in hemodialysis patients is associated with more intermuscular adipose tissue in paraspinal muscle.

## Introduction

Patients on maintenance hemodialysis (MHD) suffer from a variety of symptoms, with fatigue being one of the most disruptively, negatively impacting their daily activities and quality of life [1]. Sixty percent to seventy-five percent of people with chronic kidney disease (CKD) report fatigue, with up to a quarter experiencing severe symptoms [2]. Despite advancements in clinical care, fatigue continues to be a common complaint among MHD patients. Studies also showed that fatigue is correlated with mortality in kidney failure patients [3, 4].

The mechanisms for fatigue in MHD patients are unclear, previous work has demonstrated that fatigue was associated with demographic characteristics, comorbidities, psychological factors (e.g., depression) and laboratory variables [5–8]. Pathophysiology of muscular fatigue has been considered to be involved in the cause of fatigue in MHD patients [9]. Body composition and pathophysiology of muscular fatigue are an interacting process, but the association and mechanism are not clear. Thus, we try to elucidate if body composition is an influencing factor of fatigue. Relationship between fatigue and body composition has been studied among healthy people or patients with cancer, with mixed results [10–12]. Only one study showed that fatigue had no relationship with phase angle, an indicator of integrity of the cell membrane and intercellular space, which reflect on cell health, tested by bioimpedance electrical analysis among MHD patients [13, 14].

Body composition can be determined using a variety of methods, including dual-energy X-ray absorptiometry (DXA), bioelectrical impedance analysis (BIA), and quantitative computed tomography (QCT). Comparing with others, QCT can evaluate the fat in the skeletal muscle tissue precisely. In addition, because the QCT calibration phantom includes both fat and water samples, fat content can be recognized by the CT value of the tissue. Numerous prior researches have demonstrated that QCT examination is a reliable tool for determining the human body's composition [15, 16]. Intermuscular adipose tissue (IMAT) has been associated with lower muscle strength, power, and quality, chronic inflammation as well as impaired glucose tolerance in old adults [17–19].

Paraspinal muscle fat infiltration is more likely to be obtained by dialysis patients during chest CT or abdominal CT than thigh muscle fat infiltration, so paraspinal muscle fat infiltration is used here. Besides, multiple studies have examined skeletal muscle segmentation at various thoracolumbar levels, with the L3 monolayer being the most frequently referenced [20, 21].

Handgrip strength (HGS) is associated with overall muscle function and physical performance, particularly in populations with chronic illnesses such as hemodialysis patients. HGS serves as a simple yet reliable indicator of muscle strength and function, and its assessment can provide valuable insights into the functional status of patients undergoing hemodialysis [22].

Previous studies have shown that iron deficiency and circulating albumin are associated with fatigue among different group of patients [23, 24]. Thus, we determined the relationship between blood parameters and fatigue in MHD patients.

The purpose of this study was to investigate the relationship between fatigue and body composition parameters, HGS and biochemical blood parameters among MHD patients.

## Patients and methods

### Patients

This is a cross-sectional survey conducted from July 2022 to August 2022 in a single hemodialysis center. Inclusion criteria were: age > 18 years; end-stage renal disease on chronic hemodialysis therapy duration > 3 months. Exclusion criteria included patients having a clinical or laboratory diagnosis of malnutrition, comorbidities including active malignant malignancy, and clinical instability necessitating hospitalization. Each patient was treated with high-flux polysulfone dialyzers for an average of four hours. The conduct of this survey was reviewed and approved by the Ethics Committee of a hospital. Prior to participation, all patients read and signed an informed consent form.

### Measurements

In the survey, participants' age, gender, and dialysis therapy duration, comorbidities were collected. Hemoglobin (Hb), intact parathyroid hormone (iPTH), serum albumin and other clinical assay indexes were tested by fasting blood (a minimum of 8 h without eating) in the morning and predialysis at the accredited Chemical Laboratory. The parameter of hemodialysis adequacy, namely, Kt/V (urea), was calculated according to the Kidney Disease Outcomes Quality Initiative (KDOQI).

### Fatigue assessment

Functional assessment of Chronic Illness Therapy-Fatigue (FACIT-F) scale was used to assess fatigue of HD patients during their intradialysis period. The 13-item questionnaire examines self-reported fatigue and its impact on daily activities and function shown in Table 1. The scale consists of five points (4 = not at all; 3 = a little bit; 2 = somewhat; 1 = quite a bit; and 0 = very much). All items contribute equally to the total score. The scale ranges from 0 to 52, with 0 representing the worst possible score and 52 representing no fatigue. We divided the subjects into 2 groups [FACIT-F scores ≤ 34 (significant level of fatigue) versus > 34 (non-fatigued)] based on the previous cancer-related fatigue [25, 26].

**Table 1:** FACIT-F questionnaire

Item	General question
1	I feel fatigued
2	I feel weak all over
3	I feel listless
4	I feel tired
5	I have trouble starting things because I am tired
6	I have trouble finishing things because I am tired
7	I have energy
8	I am able to do my usual activities
9	I need to sleep during the day
10	I am too tired to eat
11	I need help doing my usual activities
12	I am frustrated by being too tired to do the things I want to do
13	I have to limit my social activity because I am tired

*FACIT-F* Functional Assessment of Chronic Illness Therapy – Fatigue

### Measurement of the lumbar bone mineral density

The original QCT images of the lumbar spine were acquired using a Siemens 64-slice force CT scanner after dialysis. The initial lumbar spine volumetric data were transmitted to a QCT Pro workstation (Mindways, Austin, USA) for bone mineral density and body composition analysis. Two musculoskeletal radiologists independently evaluated the bone mineral density (BMD). On the median plane of the L1, L2, and L3 vertebrae, the regions of interest (ROI) were automatically outlined. The ROI was manually modified to exclude cortical bone around the vertebral body. The BMD values of the L1, L2 and L3 vertebrae were acquired, and the mean BMD value was then determined using these data. As the American College of Radiology defined, BMD > 120 mg/cm^3^ was considered normal bone density, 80 ≤ BMD 120 ≤ mg/cm^3^ was considered osteopenia, and BMD < 80 mg/cm^3^ was considered osteoporosis, which is also the criterion suggested by the latest Chinese expert consensus [27].

### Measurement of L3 paraspinal muscles and IMAT

CSA in the L3 paraspinal muscle is one of the most widely used parameter to quantitatively estimate muscle mass [28, 29]. Two other musculoskeletal radiologists separately measured cross-section area (CSA) of the L3 paraspinal muscles, and IMAT at the mid-vertebral level slice of L3 while blinded to the clinical information and BMD values of patients. The ROI were drawn as large as possible to encompass the entire muscle without exceeding its shape. IMAT was characterized in our study as an ectopic fat deposit within the muscles and beneath the fascia according to Addison's review [30]. IMAT and CSA were calculated semiautomatically by the commercial software QCT Pro6.1(Mindways, Austin, USA). In contrast to absolute HU values derived from CT images in the literature [31], HU measurements derived from QCT imaging are converted into tissue (i.e., Muscle and fat) densities by scanning the subject alongside a calibration phantom containing standards representing known bone and soft tissue densities. To get a more standardized measurement, the L3 SMI was calculated as the total CSA of the skeletal muscle adjusted by the square of the height, On the basis of measurements of muscle CSA and IMAT CSA, the IMAT% (CSA_IMAT_ / CSA_IMAT_ + CSA_muscle_) was computed to indicate fatty infiltration of muscle [32].

### Handgrip strength

Using a dynamometer (WCS-100, Nantong, China), the standard outcome of combined HGS was measured on dialysis day. Patients were instructed to squeeze a dynamometer with the elbows flexed at a 90-degree angle and in standing position which could produce moderately larger HGS outcomes in comparison with sitting [33]. Patients were asked to exhale and exert maximal grip for 3 s every repeat, testing with their dominant hand. Then, there was a 60-s interval between each measurement. The highest HGS (kg) was recorded.

### Determination of sample quantities

In accordance with TRIPOD Guide, the sample quantity of a clinical predictive model is calculated according to the calculation methodology of events per variable (EPV), i.e., the guidelines for the number of positive events corresponding to each variable are recommended to meet at least EPV = 10.The predicated model constructed in this study contains 3 variables with the required number of positive events (fatigue patients) of at least 10*3 = 30 cases. Since the proportion of fatigue patients was 34:92*100% = 37%, the samples required could be met in 30:37% to 81 cases. Based on the available data of the study, the final sample was 92 cases.

### Statistical analysis

The statistical analysis was performed using SPSS 19. Data were presented as mean and SD for normally distributed data, median and interquartile range for non-normally distributed data, or percentage frequency for binary data. Group comparisons were conducted using *t* test for normally distributed data, *Mann–Whitney U* test for non-normally distributed data, or *chi-squared* test for binary data, as appropriate. The characters which are different between two groups (*P* < 0.05) step into correlation analysis. Pearson correlation analysis was utilized for normally distributed data, and Spearman correlation analysis was utilized for non-normally distributed data. A univariate logistic regression analysis was conducted. The variables selected by univariate logistic regression analysis (*P* < 0.05) were then further screened using the backward stepwise multivariable regression method to predict the risk factors for fatigue. *P*-values less than 0.05 were considered statistically significant.

## Results

### Clinical characteristics of all the subjects

In total, 92 patients were included, among which 52.5% were male. The mean age of patients was 57.8 (SD = 12.5) years. The prevalence of fatigue was 37%, abnormal bone density was 43.4% (*n* = 40). Patients were divided into fatigue group (FATIC-F score ≤ 34) and non-fatigue group (FACIT-F > 34). Compared with non-fatigued patients, fatigued patients are advanced age, and have lower serum phosphorus. Moreover, fatigued patients have the higher IMAT%, lower bone mineral density, lower handgrip strength comparing with the non-fatigued patients (Table 2). However, there was no difference between two groups in Hb, dialysis therapy duration, and SMI.

**Table 2:** Patients demographics and body composition measured by quantitative computer tomography (QCT)

Variables	Overall (n = 92)	Fatigued (n = 34)	Non-fatigued (n = 58)	t/z/χ^2^	*P* values
Male (%)	48 (52.5)	17 (50)	31 (53.4)	− 0.31	0.75
Age (years)	57.8 ± 12.5	64.3 ± 11.6	54.0 ± 11.5	4.12	< 0.001
Comorbidities					
Hypertension (%)	74 (80.5)	26 (72.5)	48 (82.8)	0.539	0.463
Diabetes (%)	28 (30.4)	14 (41.2)	14 (24.1)	2.939	0.086
Cardiovascular disease (%)	20 (21.7)	10 (29.4)	10 (17.2)	1.866	0.172
Dialysis therapy duration (months)	81.2 ± 46.4	80.1 ± 38.9	81.7 ± 50.6	− 0.16	0.87
Vascular access				2.43	0.11
Arteriovenous fistula	86 (93.5)	30 (88.2)	56 (96.6)		
Central venous catheter	6 (6.5)	4 (11.8)	2 (3.4)		
Systolic blood pressure	140 ± 25	139 ± 27	140 ± 24	0.18	0.85
Diastolic blood pressure	81 ± 13	77 ± 13	83 ± 13	1.75	0.08
Kt/V	1.60 ± 0.35	1.58 ± 0.38	1.62 ± 0.34	0.41	0.66
BMI (kg/m^2^)	23.4 ± 3.6	24.6 ± 2.8	22.8 ± 3.8	2.10	0.03
Hb (g/L)	110.3 ± 15.6	113.1 ± 13.3	108.7 ± 16.7	1.32	0.19
Alb (g/L)	38.6 ± 4.8	37.9 ± 5.5	39.0 ± 4.4	− 1.05	0.29
ALP (U/L)	90 (72,128)	87 (77, 107)	94 (67, 134)	− 0.51	0.60
BUN, mmol/L	23.7 ± 6.4	22.4 ± 5.7	24.5 ± 6.8	1.49	0.13
Creatinine, umol/L	867 ± 211	807 ± 206	902 ± 208	2.08	0.04
Uric acid, mmol/L	430 ± 95	398 ± 89	449 ± 94	2.50	0.01
Total cholesterol, mmol/L	4.2 ± 0.9	3.9 ± 0.8	4.3 ± 1.0	1.73	0.08
Triglyceride, mmol/L	1.8(1.2, 3.0)	1.9(1.2, 2.6)	1.8(1.1, 3.2)	0.61	0.54
High-density cholesterol	0.9(0.7, 1.2)	0.8(0.7, 1.1)	1.0(0.8, 1.2)	2.19	0.03
Low-density cholesterol	2.2 ± 0.8	2.0 ± 0.5	2.3 ± 0.9	1.84	0.06
Corrected serum Ca (mmol/L)	2.2 (2.0, 2.4)	2.2 (2.1, 2.4)	2.1 (2.0, 2.4)	− 1.37	0.17
Serum P (mmol/L)	1.8 ± 0.5	1.7 ± 0.5	1.9 ± 0.5	− 2.37	0.02
iPTH (pg/ml)	335 (185, 520)	406 (179, 527)	288 (185, 518)	− 0.71	0.47
Ferroprotein (ug/L)	126 (52, 283)	103 (71, 269)	143 (49, 301)	− 0.15	0.88
Transferrin (g/L)	1.9 (1.6, 2.2)	1.8 (1.5, 2.1)	1.9 (1.6, 2.3)	− 1.30	0.19
SMI (cm^2^/m^2^)	15.6(13.9, 17.6)	15.3(14.2, 17.6)	15.7(13.5,17.7)	− 0.12	0.90
BMD (mg/cm^3^)	127.8 ± 42.8	116.7 ± 48.3	134.2 ± 38.4	− 1.87	0.06
IMAT %	8.2(6.3, 12.4)	9.7 (7.6, 15.9)	7.1 (4.4, 11.0)	− 3.86	< 0.001
β2-microglobulin (mg/L)	29.7 (23.3, 35.2)	30.6 (25.9, 35.2)	29.6 (22.6, 34.9)	− 0.50	0.61
Use of phosphate binder (%)	80(86.9)	29(85.2)	49(87.9)	0.874	0.536
Handgrip strength (kg)	23.0 ± 8.4	18.6 ± 6.3	25.1 ± 8.6	− 3.48	< 0.001

*BMI* body mass index, *Hb* hemoglobin, *Alb* albumin, *Ca* calcium, *P* phosphorus, *iPTH* intact parathyroid hormone, *SMI* skeletal muscle index, *BMD* bone mineral density; IMAT, intermuscular adipose tissue, *ALP* alkaline phosphatase

### Factors associated with fatigue in hemodialysis patients

There was a positive correlation between handgrip strength and FACIT-F score. Age, (*r* = − 0.411, *p* < 0.001), IMAT % (*r* = − 0.424,* p* < 0.001) negatively associated with FACIT-F score. There was no correlation between BMD and FACIT-F score (Table 3). A multivariate regression analysis was conducted to identify factors related with fatigue. As shown in Table 4, lower serum phosphorus (*OR* = 0.333, *95%CI* [0.122–0.913], *P* = 0.033), higher IMAT% (*OR* = 1.147, *95%CI* [1.012–1.301], *P* = 0.032), old age (*OR* = 1.059, *95%CI* [1.009–1.113], *P* = 0.021) are associated with a high risk of fatigue.

**Table 3:** Correlations between FACIT-F score and continuous variables

Variables	*r*	*P* value
Age	− 0.411	< 0.001
BMI	− 0.079	0.47
Serum phosphorus	0.200	0.056
Creatinine, umol/L	0.287	0.006
Uric acid, mmol/L	0.234	0.026
High-density cholesterol, mmol/L	0.141	0.188
IMAT%	− 0.424	< 0.001
Handgrip strength	0.448	< 0.001

*BMI* body mass index, *IMAT* intermuscular adipose tissue

**Table 4:** Binary logistic regression analysis of risk factors for fatigue

Factor	Univariate analysis			Multivariate analysis		
	OR	95%CI	*P*-value	OR	95%CI	*P*-value
Age	1.083	1.036–1.132	< 0.001	1.059	1.009–1.113	0.021
Creatinine, umol/L	1.002	1.000–1.005	0.046	–	–	–
Uric acid, mmol/L	1.006	1.001–1.011	0.018	–	–	–
Serum phosphorus, mol/L	0.379	0.165–0.874	0.023	0.333	0.122–0.913	0.033
IMAT%	1.622	1.247–2.110	< 0.001	1.147	1.012–1.301	0.032
Handgrip strength	0.896	0.835–0.960	0.002	–	–	–

*IMAT* intermuscular adipose tissue, *CI* confidence interval, *OR* odds ratio

## Discussion

Fatigue is a prevalent symptom of MHD patients that imposes a tremendous load on the individual's life, diminishes their quality of life, and causes enormous financial losses [1]. Currently, there is no consensus regarding the best methods for measuring fatigue in patients with MHD. In our study, FACIT-F was used given that this questionnaire is fatigue-specific and uncomplicated to acquire from MHD patients [2]. Besides, studies also showed that for patients receiving hemodialysis, FACIT-Fatigue exhibited acceptable validity and reliability [34, 35]. However, some of the items may also be influenced by other factors, time constraints at work and a lack of appetite may contribute to sleep and eating difficulties, rather than simple fatigue. Besides, for MHD patients’ vascular access arteriovenous fistula or central venous catheter can also be factors that constrain their usual activities. Thus, some faults exist in this questionnaire for MHD patients, a more suitable questionnaire was needed for HD patients to evaluate fatigue.

In our study, the prevalence of fatigue is 37%, the rate is lower compared with other studies. The cut-off setting of FACIT-F scale may result that. Comparing with the cutoff of ≤ 44 used in other studies [36, 37], the cutoff of ≤ 34 is stricter to identifying fatigue. Besides, different from other diseases, MHD patients have unique temporal patterns of fatigue [38] because of the hemodialysis procedure. The timing of data collection and patient characteristics at the time of assessment may also impact fatigue prevalence rates. In our study, patients finished questionnaire during dialysis time, they do not participate in questionnaire surveys when they experience severe symptoms. Longitudinal studies capturing fluctuations in fatigue over time may provide a more comprehensive understanding of its prevalence and associated factors among hemodialysis patients. Understanding the mechanism of fatigue among MHD patients is crucial for treatment of fatigue. To date, the variables and underlying mechanisms have been inadequately characterized. In other conditions, fatigue is correlated with skeletal muscle mass [23]. Decreasing skeletal muscle mass may be the primary cause of a sense of exhaustion, general weakness, and energy deficiency, which can result in physical fatigue [39]. In MHD patients, a study showed that fatigue is associated with extracellular water excess, and patients with decreased muscle mass were less likely to report fatigue, probably because they accepted their physical limitations [40].

In the present study, we found patients with higher IMAT were more likely to report fatigue. Previous research has shown that people on MHD tend to accumulate more adipose tissue in their muscle [41]. A recent study also show that severity of kidney disease was found to correlate with progressive intramuscular adipose tissue accumulation [42].

The quality and performance of muscles may be impacted by intramuscular adipose tissue, which is also linked to impaired physical performance [43]. However, there was no relationship between SMI and self-reported fatigue. Moreover, a study reported that in the diagnosis of sarcopenia, fat infiltration of skeletal muscle demonstrated greater predictive value than L3 SMI [36]. Therefore, we can reach that fatigue is correlated with sarcopenia, but SMI is not sensitive.

Potential causes of lipid accumulation in muscle include high plasmatic fatty acid concentrations (oversupply), an imbalance between oxidation and uptake of fatty acids [44]. What is the potential mechanism underlying the fatty infiltration in skeletal muscle even with lipid metabolism parameters justification? On the other hand, lipid metabolism occurs mostly in mitochondria, suggesting that mitochondrial malfunction may contribute to intramuscular adipose tissue formation, a study found that a strong link between mitochondrial function and intramuscular adipose tissue [42]. Moreover, increased levels of toxic lipid metabolites and adipose-derived cytokines affect the signaling and mitochondrial function of muscle cells, which contributes to the loss of force-producing capacity [45]. Mitochondrial malfunction and intramuscular adipose tissue formation is also an interacting process. Further mechanistic study is needed to elucidate the connection between mitochondrial dynamics and fatigue in MHD patients, despite evidence showing that altered mitochondrial dynamics impair muscular performance. A recent study showed that exercise efficiency was found to be related to mitochondrial capacity [46]. Further, a study found that physical inactivity is connected with a high percentage of fat in the multifidus muscle [47], which supporting the idea that exercise may be a therapeutic way for fatigue. Furthermore, small clinical trials have demonstrated the advantages of physical activity in improving fatigue in patients on hemodialysis. Interventions involved intra-dialytic exercise [48, 49] and walking sessions on non-dialysis days [50]. However, implementing a workout routine requires thought. Fatigue is a common reason individuals avoid exercise [51, 52]. The studies emphasized patients' choice of exercise modality and timing, safety of HD vascular access during exercise, and demand for attainable regimens [52]. A trainer may be necessary for optimal involvement in intradialytic exercise. Without a kinesiologist, participants were more likely to decline the session and report being too weary to engage [51]. Professional presence may motivate adherence to fitness treatments.

High serum phosphorus has always been a risk factor of cardiovascular disease for MHD patients. Interestingly, in our study we found that high serum phosphate is associated with lower risk of fatigue. A previous study shows that serum phosphate is negatively associated with low handgrip strength in patients with advanced chronic kidney disease [53], which is opposite with our study. The possible reason is that the different population. There are substantial variations in fatigue across CKD stages and fatigue in HD patients. According to previous literatures, to meet cells metabolic needs not only in the cytoplasm but also at the subcellular level, cells should be able to sense intracellular and extracellular inorganic phosphate (P) levels. To produce ATP, mitochondria rely on P availability for oxidative phosphorylation and the electron chain reaction. Thus, for skeletal tissue, it is pretty important to maintaining balance of P [54]. Internal environment disturbance in MHD patients may lower the cells’ ability to sense extracellular ambient P, which presents lower serum P are associated with fatigue. However, more mechanism studies are needed to prove how the cells change their ability to sense extracellular ambient P.

Several limitations need to be addressed. First, the sample size was small in this cross-sectional study with a single center. Second, our study used the recognized cut-off point for FACIT-F scale. Given that the FACIT-F cut-off value for diagnosing fatigue may vary amongst populations, additional research is required to discover the FACIT-F cut-off value in the MHD patients.

## Practical application

This study is first to describe the association between fatigue and fatty infiltration of paraspinal muscle among hemodialysis. It will be valuable for healthcare professionals in identifying fatigue risk factors and burdens, and devise measures to alleviate this symptom in hemodialysis patients.

## Data Availability

All data generated or analyzed during this study are included in its supplementary information files.

## Abbreviations

CKD: Chronic kidney disease
CSA: Cross-section area
BIA: Bioelectrical impedance analysis
BMD: Bone mineral density
DXA: Dual-energy X-ray absorptiometry
EPV: Events per variable
FACIT-F: Functional Assessment of Chronic Illness Therapy-Fatigue
Hb: Hemoglobin
HGS: Handgrip strength
IMAT: Intermuscular adipose tissue
iPTH: Intact parathyroid hormone
MHD: Maintenance hemodialysis
P: Phosphate
QCT: Quantitative computed tomography
ROI: Regions of interest
SMI: Skeletal muscle index
